# Adoption of digital technologies and backshoring decisions: is there a link?

**DOI:** 10.1007/s12063-021-00202-2

**Published:** 2021-07-28

**Authors:** Bart Kamp, Juan José Gibaja

**Affiliations:** Deusto University, Orkestra-Basque Institute of Competitiveness and Deusto Business School, Kalea Mundaiz 50, 20012 Donostia/San Sebastian, Spain

**Keywords:** Industry 4.0, Backshoring, Production location, International business, Global value chains, D22, F23, L23, L60

## Abstract

The present paper assesses whether the adoption of Industry 4.0 technologies can be related to backshoring. It does so by -firstly- investigating the implementation of such technologies by industrial firms with foreign production plants, the experiences and intentions of these firms regarding the location of production activities, and -secondly- by analyzing backshoring cases among them.

It finds that backshoring is a rare phenomenon, and it is questionable whether there is a correlation, left alone causality, between the adoption of digital technologies in home-based manufacturing sites and backshoring hitherto. And while the future may hold more backshoring movements in store, they may not be primarily due to the adoption of Industry 4.0 technologies at home-based plants. Instead, other (foreign) location-specific factors seem to have greater weight in the decision-making processes around backshoring operations. I.e., deteriorating sales forecasts in offshore places where firms have production activities, increases in institutional uncertainty in such places, rationalization of global production apparatuses, and/or a lack of possibilities to deploy foreign manufacturing activities and output for third markets. Also against the backdrop of events like the outbreak of Covid19 and the uncertainty-raising effect it has on international business, the trade-off between producing off-shore or bringing manufacturing activities back home is not likely to depend on technology adoption levels at home and abroad either.

## Introduction

A growing body of literature is looking into the impact of digital technologies on the course of international business (Van Tulder et al. 2018). As part of this stream of research, there is growing scholarly interest in investigating how advanced manufacturing technologies influence the geographical location of production activities. In this regard, Bals et al. (2015) and Fratocchi et al. (2014) study to what extent the application of productivity-enhancing technologies in developed countries may facilitate the backshoring of production activities from emerging economies.

Similarly, Ancarani and Di Mauro (2018, 2019) as well as Fratocchi and Di Stefano (2019) examine whether the adoption of Industry 4.0 bears any relationship to reshoring decisions. In addition, also Dachs et al. (2019) and Chiarvesio and Romanello (2018) explore the link between Industry 4.0 and backshoring of production activities.

Dachs et al. (2019) assert that Industry 4.0 and local manufacturing strategies could have a substantial impact on backshoring in upcoming years. Consequently, they call for new insights to evaluate the relationship between backshoring of production activities and investment in Industry 4.0 technologies at European manufacturing firms.

Despite the importance of assessing whether the uptake of advanced manufacturing technologies influences the backshoring phenomenon, when reviewing the corresponding state-of-the-art, it turns out that publications referring to the concept of Industry 4.0 either lack a clear operationalization of it (Chiarvesio and Romanello 2018) and/or reduce it to a limited set of representative technologies (Dachs et al. 2019; Ancarani and Di Mauro 2018, 2019; Fratocchi and Di Stefano 2019).

Instead of the former, the present paper adheres to a comprehensive breakdown of Industry 4.0 into a broad range of technologies, as introduced by Rüßmann et al. (2015).^1^ The former technology breakdown is probably the one that has received most following among academic scholars (see e.g. Gilchrist 2016; Chiarvesio and Romanello 2018; Saucedo-Martinez et al. 2016; Alcacer and Cruz-Machado 2019; Hernandez-de-Menendez et al. 2020; Stentoft et al. 2020). Hence, it serves as our point of reference. Afterwards, we use this breakdown to assess whether and how the adoption of the respective technologies is intertwined with backshoring dynamics.

To that end, the rest of the paper is structured as follows: Sect. 2 outlines the research context and reviews the extant literature, Sect. 3 introduces the methods and data used to conduct our research, Sect. 4 presents the research findings, Sect. 5 discusses the results obtained, Sect. 6 discusses the implications, and Sect. 7 highlights the limitations of the results and insights generated by our research.

## Research context

The term Industry 4.0 refers to a family of technologies that entail the use and coordination of information, automation, computation and sensing devices for the sake of advanced manufacturing activities (Acatech 2015; Posada et al. 2015; Kagermann 2015).

From an international business and production operations perspective, the adoption of these technologies can influence the decisions regarding the (future) location of production activities (Gray et al. 2013; Chiarvesio and Romanello 2018). As such, the adoption of digital technologies could provide a counterweight against the process of offshoring and the articulation of supply or value chains on a global level. That is: if they are unilaterally -or more intensely- adopted in the home base of companies that have previously offshored production. The way that Industry 4.0 technologies can impact on location decisions, and could have a backshoring effect, stems roughly from the following mechanisms. They can:

- equalize cost levels between high- and low-cost countries (Tate 2014; Bals et al. 2016),
- generate benefits from bringing production activities closer to the end market, thus endowing production processes with superior possibilities in terms of planning flexibility and delivery compliance (Kinkel and Maloca 2009; Lu 2017; Johansson and Olhager 2018; Dachs et al. 2019)
- create synergies through (re)coupling manufacturing to a company’s hotspots for R&D, design and product development (Brettel et al. 2014; Rezk et al. 2016; Fratocchi et al. 2014; Lu 2017; Di Mauro et al. 2018; Johansson et al. 2019),
- and/or raise quality standards and customization possibilities for products and operations (Stentoft et al. 2016a, b; Johansson et al. 2019; Ancarani et al. 2019; Dachs et al. 2019).

Consequently, scholars have started to explore the so-called backshoring or reshoring phenomenon (Kinkel 2012, 2014; Gray et al. 2013; Di Mauro et al. 2018).

While research on Industry 4.0, on the one hand, and backshoring, on the other, is growing; publications that look at both simultaneously are still rare, with Chiarvesio and Romanello (2018), Ancarani and Di Mauro (2018, 2019), Dachs et al. (2019) and Fratocchi and Di Stefano (2019) being the exceptions. As such, this field of research is still in a nascent phase (Edmonson and McManus 2007). A typical feature of research domains that are in the early stages is that they face several loose ends. Against this backdrop the present paper sets out to make contributions in the following areas:

1. Adopt a neater approach to Industry 4.0 than has been done so far around backshoring analyses. Instead of treating it as an umbrella term or in a *pars pro totum* manner, propose a comprehensive breakdown into underlying technologies for the sake of research in the backshoring realm (Sect. 2.1).
2. Build on research that points at the relevance of certain technologies for specific backshoring rationales, by examining such relevance and by broadening the base of Industry 4.0 technologies that can be looked into (Sect. 2.2).
3. Build on research that looks beyond technology as a factor for relocation decisions, to provide relief to the role that Industry 4.0 technologies play in such decisions (Sect. 2.3).

### Operationalization of industry 4.0 adoption

Extant research on Industry 4.0 and backshoring has delivered thus far rather inconclusive results. For starters, because the few publications available do not lead to the same conclusions. Rather, they have produced opposite results.

To begin with, the results of Dachs et al. (2019 p. 1) support the idea that the adoption of advanced manufacturing technologies in the home base stimulates backshoring: “Descriptive statistics as well as regression results indicate a positive correlation between the adoption of Industry 4.0 technologies and companies’ backshoring propensity.”

However, there is counterproof coming from Ancarani et al. (2019 p. 368) who state that: “to date backshoring has largely taken place without investment in new technologies” and from Chiarvesio and Romanello (2018 p. 371) when they state that they “cannot identify a clear and direct relationship among investments in Industry 4.0 technologies and international activities, neither declared by the companies, nor indirectly identified during data collection.” Also Müller et al. (2017 p. 165) conclude that “the general estimation for the importance of reshoring of Industry 4.0 remains questionable”.

In turn, Fratocchi and Di Stefano (2019) offer mixed results, indicating that automated machinery technologies are relevant for backshoring in case they are applied to repatriation of production activities that were previously offshored for cost motives. Conversely, as regards 3D printing (or additive manufacturing) they only find very weak indices that this technology plays a role in backshoring decisions. Interestingly, Ancarani and Di Mauro (2018) argue that robotics (rather close to automated machinery) is not really an enabler for reshoring, while they argue at the same time that additive manufacturing can be an effective enabler of reshoring moves.

Evidently, there is a big variation in the sources from which these scholars depart (European Manufacturing Survey, Univaq Manufacturing Reshoring dataset focused either on European backshoring cases only or on European and North American cases or case studies) and the number of instances that they work with: 1705 in the case of Dachs et al. (2019), 495 on behalf of Ancarani et al. (2019), 840 in the case of Ancarani and Di Mauro (2018), 816 on behalf of Fratocchi and Di Stefano (2019), 50 for Müller et al. (2017) and 16 in the case of Chiarvesio and Romanello (2018). So do the data analysis techniques deployed: regression analysis in the case of Dachs et al. (2019) and Ancarani et al. (2019), descriptive statistics in the case of Müller et al. (2017), Ancarani and Di Mauro (2018) as well as Fratocchi and Di Stefano (2019), and qualitative analyses in the case of Chiarvesio and Romanello (2018). Finally, there is also a broad variety regarding the data character behind the sources employed: primary data from own survey in the case of Müller et al. (2017) and Dachs et al. (2019), whereas Ancarani and Di Mauro (2018), Ancarani et al. (2019 and Fratocchi and Di Stefano (2019) employ secondary data, and Chiarvesio and Romanello (2018) recur to cases for their primary data.

The former helps to explain why the “results of these studies are difficult to compare” (Müller et al. 2017 p. 169), but it does not change the inconsistency among the different findings.

Most importantly perhaps, while there is consensus on what backshoring or reshoring stands for and how it can be segmented (following e.g. seminal frameworks by Gray et al. 2013, p. 28),^2^ the same can not be said about Industry 4.0. That is: from a perspective of the technologies concerned. Only two of the seven extant publications that examine the phenomenon of backshoring from an Industry 4.0 angle consider the entire set of technologies from Rüßmann et al. (2015). These are Chiarvesio and Romanello (2018) and Stentoft et al. (2020). The remaining publications apply a rather selective or open approaches regarding the Industry 4.0 technologies that they focus on. Among the selective approaches, we find Dachs et al. (2019) who look at eight technologies of which only three bear a clear relationship with the framework for Industry 4.0 as introduced by Rüßmann et al. (2015).^3^ The other ones^4^ are indeed digital technologies or exponents thereof, but they are “exogenous” to the Industry 4.0 framework of reference that is used for this paper. Ancarani and Di Mauro (2018), for their part, consider three technologies, of which two can be considered “endogenous” to Industry 4.0.^5^ Fratocchi and Di Stefano (2019) look at four types of technology, of which two adhere to the Rüßmann et al. (2015) framework.^6^ Among the open approaches, Ancarani et al. (2019, p. 365) use “keywords such as Industry 4.0, robotics, automation, additive manufacturing, 3DP, smart manufacturing, digitalization, advanced manufacturing, etc.” to assess whether new technology adoption can be related to backshoring. However, this seems to have served for detecting cases of backshoring where some kind of advanced manufacturing technology was involved, instead of a one-by-one probing of the involvement of specific Industry 4.0 technologies in backshoring cases. Similarly, Müller et al. (2017), do not break down Industry 4.0 into specific technologies for their survey, and just use Industry 4.0 as an umbrella term to ask questions to their respondents. Moreover, they treat Industry 4.0 and reshoring in a rather disjointed manner, without manifest attempts to interlink the two concepts.

Finally, Chiarvesio and Romanello (2018 p. 361) and Stentoft et al. (2020)^7^ look at the entire range of Industry 4.0 technologies, based on the classification of Rüßmann et al. (2015).^8^ However, they do not come to concrete verdicts as to how the respective technologies relate to backshoring behavior. Instead, they come up with more aggregate statements and insights.

### Relevance of respective digital technologies for specific backshoring rationales

Ancarani and Di Mauro (2018), as well as Fratocchi and Di Stefano (2019), postulate certain "production strategy-technology preference" combinations in their publications (see Table 1).

**Table 1:** Technologies as levers for backshoring according to specific production strategies

Production strategy	Corresponding technologies according to Ancarani and Di Mauro (2018)	Corresponding technologies according to Fratocchi and Di Stefano (2019)
Cost-oriented	Robotics, automated machinery	Automated machinery
Flexibility-oriented	Robotics, automated machinery	Robotics, automated machinery
Quality-oriented	3D printing	3D printing, CPS

own elaboration

I.e., Fratocchi and Di Stefano (2019) relate « automated machinery» to backshoring that focuses on cost savings in the first place. As such, this type of machinery tends to be connected to production activities that were offshored to seize low cost advantages overseas. In addition, they argue that investments in automated machinery can form part of backshoring decisions that are flexibility-oriented, as they allow firms to adapt products for different customer types.

Conversely, they attribute a quality-orientation feature to 3D printing: when relocation to the home country is aimed at product upgrade, it is indicated to leverage 3D printing.

In a similar style, Ancarani and Di Mauro (2018) relate robotics (and automated machinery) to cost-oriented reshoring, and 3D printing and Cyber-Physical Systems (CPS) to quality-oriented reshoring.

Finally, both Fratocchi and Di Stefano (2019) and Ancarani and Di Mauro (2018) attribute flexibility virtues to robotics and automated machinery, and view them as appropriate for flexibility-oriented reshoring decisions, consequently.

Stentoft et al. (2020) focus on backshoring for cost reasons, but do not look into specific Industry 4.0 technologies and the distinct role they may play in this regard.

### Contextualizing the role of technology in backshoring decisions

While the use of Industry 4.0 technologies may have explanatory power for understanding backshoring movements, clearly there can also be other considerations or factors that add to the explanation of backshoring moves. In other words, when discovering that the adoption of Industry 4.0 technologies coincides or is intertwined with backshoring decisions, this does not entail that it constitutes the main or only driver behind such decisions.

In this sense, Gray et al. (2013), Johansson et al. (2019) and Dachs et al. (2019) argue that offshoring or reshoring decisions can be reviewed through the OLI paradigm (Dunning 2001)^9^ and Transaction Cost Economics (Williamson 1985). Notably by looking at a company’s (in)ability to exploit its “organization-specific advantages” (like a competitive product or a sought-after recipe or brand); at the “location-specific (dis)advantages” of the offshore location where production activities are undertaken at a given moment, from e.g. a market access,^10^ technology, skilled labour and suppliers availability; at the “behavioral (un)certainties” with regard to the demand side and of personnel in the host country; at the “environmental (un)certainties” *sur place* in the sense of institutional (in)stability, (un)favorable fiscal and international trade regimes and (lack of) respect for (intellectual/private) property rights; and at the possibilities to coordinate and integrate offshore assets within the overall business of a multinational (cfr. “internalization (dis)advantages”). Whereas Johansson et al. (2019) find through exploratory factor analysis that access to technology -as part of their development “bundle”- is important for backshoring decisions, and the descriptive results from Dachs et al. (2019) reveal that backshoring occurs most frequent in high-technology industries where firms may react fast to changing technology conditions across the globe, Gray et al. (2013) do not refer to technology as a possible factor for backshoring at all.

## Methods

### Empirical setting

The empirical research setting from which we draw data is the Basque Country. We posit that it provides a relevant testing ground since the Basque economy has an above-average reliance on industrial activities. Manufacturing activities represent almost a quarter of the economy’s gross value added, which is clearly higher than the Spanish and EU ratio (Kamp and Ruiz de Apodaca 2017). In fact, this region is a traditional industrial heartland of the Iberian Peninsula, and although it has undergone significant industrial conversion in recent decades, it has demonstrated an ongoing political commitment to maintaining industry at the center of the regional economy (Konstantynova 2017). As such, it can be expected to house an active landscape of manufacturing firms with an interest in adopting the latest production technologies, e.g. in the form of Industry 4.0.

### Research activities

Two research objectives guided the undertaken investigation, namely: 1) whether the adoption of Industry 4.0, or digital technologies, bears a relation to backshoring decisions and their rationales (bullet points 1 and 2 at the end of Sect. 2), and 2) the role of Industry 4.0 technologies among other possible factors influencing such decisions (bullet point 3 at the end of Sect. 2).

The first objective has a rather analytical (theory-testing) character, whereas the second is more exploratory (theory-developing) in nature. Hence, we adopt a two-stage research approach and organized a comprehensive survey in view of the first objective, followed by in-depth case analyses to attain the second (Dul and Hak 2008). The first stage is in line with Dachs et al. (2019) who also apply a survey to investigate the relationship between backshoring of production activities and the use of digital technologies. The second stage aligns with Joubioux and Vanpoucke (2016) and Benstead et al. (2017) who likewise conducted in-depth case studies to study reshoring decision-making processes and to explore how backshoring unfolds. The combination of surveying companies and conducting case analysis also has the advantage that it can help shedding light on eventual causalities between the adoption of Industry 4.0 technologies and backshoring dynamics.

Given that Industry 4.0 and backshoring are relatively new phenomena, it is challenging to probe whether it is Industry 4.0 adoption that leads to backshoring or the other way around (Martinsuo and Chaoji 2017). Therefore, to go beyond revealing patterns or correlations between digital technology adoption and backshoring decisions (as per the survey activities), in-depth case analyses are useful to get an idea of eventual causalities between the former and the latter (Stentoft et al. 2016a, b).

**Image 1:**
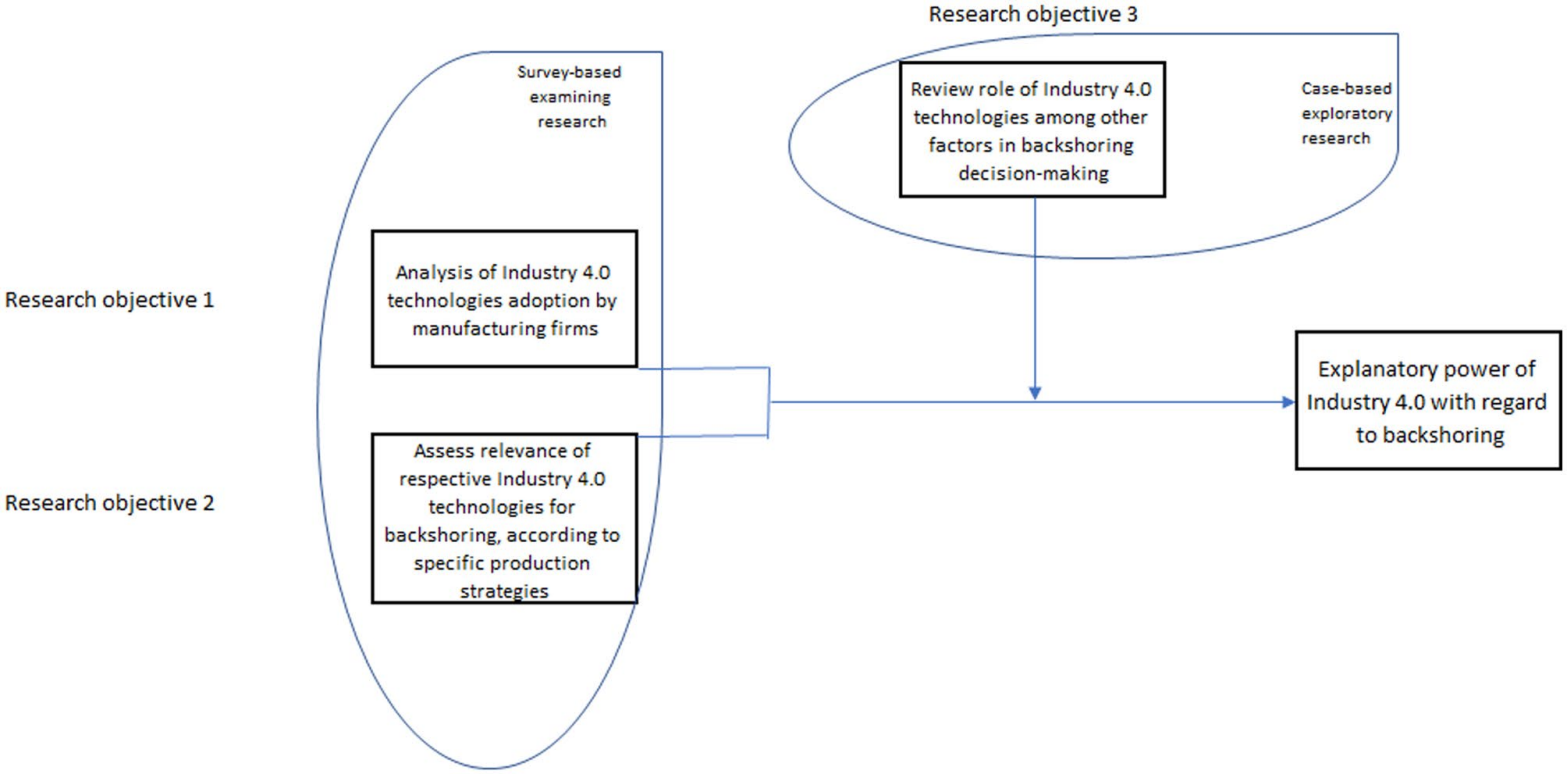
Interrelations between research objectives and methods applied. Source: own elaboration.

#### Survey-based research activities

To attain research objectives 1 and 2 (see Image 1 above), a large-scale survey was organized supported by a structured questionnaire.

For the questionnaire design and to operationalize what Industry 4.0 stands for, the Rüßmann et al. (2015) classification was used as well as the Digital Transformation Monitor of the European Commission (DTM 2018). The reason for the latter was that the survey was backed up by the Industrial Agency of the Basque Government (SPRI) who had an interest in comparing the survey results with the DTM (2018). This led to the inclusion of a list of technologies in the survey questionnaire that does not align completely with the Rüßmann et al. (2015) classification, although we did manage to cover it almost entirely by making use of a proxy and a “composite indicator”.

As for the “composite indicator”; to cover Horizontal and Vertical systems integration (HVSI), we assessed whether companies had adopted machine-to-machine (M2M) communication both at an intra-firm and inter-firm (together with clients and/or supplier) level. While this may not always be the same as what Rüßmann et al. (2015) portray by HVSI, it should come close. Moreover, it is arguably a more operational yardstick, which makes it more plausible that respondents answer correctly when asked about having implemented this concept at an intra and inter-firm level, than the platform that Rüßmann et al. (2015) refer to. Furthermore, as Chiarvesio and Romanello (2018, p. 361) explicate: M2M communication serves the purpose of HVSI. That is: manufacturing and machine data feed collaboration platforms from production to corporate, within the company, and with suppliers or clients. Consequently, we deem that our proxy is a valuable substitute for the original term.

Finally, we applied the term “robotics”, which is not necessarily the same as “autonomous robots” and can thus only serve as a proxy.

For a complete overview of the Industry 4.0 technologies that Rüßmann et al. (2015) consider, how our research dealt with those (and others), as well as the technology sets that other (backshoring) scholars considered, see Table 2.

**Table 2:** Industry 4.0 technologies considered in backshoring publications

Rüßmann et al. (2015)	Digital Transformation Monitor of the European Commission	This paper	Ancarani and Di Mauro (2018)	Dachs et al. (2019)	Fratocchi and Di Stefano (2019)	Chiarvesio and Romanello (2018)	Stentoft et al. (2020)
1-Internet of Things	X	x			Stentoft et al. (2020)	X	X
2-Cloud Technologies	X	x				X	X
3-Big Data Analytics	X	x				X	X
4-Virtual Simulation Systems		x				X	Proxy = Simulation
5-Augmented Reality		x				X	X
6-Additive manufacturing / 3D Printing	X	x	X	X	X	X	X
7-Horizontal and Vertical systems integration		Proxy = Machine-to-Machine communication in cooperation with suppliers and/or clients		Proxy = Digital exchange of data with suppliers / customers + Near real-time production control systems		x	x
8-Autonomous robots	Proxy = robotics	Proxy = robotics	Proxy = robotics		Proxy = robotics	Proxy = Autonomous and collaborative robots	x
9-Cybersecurity	X	x				X	X
Additional technologies							
Artificial Intelligence	x	x					X
Automated Machinery	x	x			X		
Cyber-Physical Systems		x	X	Proxy = Technologies for human–machine interaction			
Digital Visualization				x			
Mobile Services	X	x		Mobile / wireless devices for providing services			Mobile technologies
Product-Lifecycle Management Systems				X			
RFID and Real-Time-Location Systems (RTLS) technologies							x
Social Media	X	x					
Systems for automation of internal logistics				X			

*own elaboration*

The survey included questions to determine the participating companies’:

- Size in terms of revenue and employees
- Value chain position
- Principal products and market offerings
- Foreign branch plants
- Adoption of digital technologies
- Intentions to backshore manufacturing activities

Apart from asking the survey participants whether they had adopted any of the listed technologies during the last 3 years (Research objective 1), they were asked whether the adoption of Industry 4.0 technologies could lead to backshoring maneuvers by their company and the likelihood of taking such decisions (Research objective 2). To distinguish between the degree of certainty with which they expected this to happen, we provided respondents with the possibility of answering either: “Yes, in fact, such decisions have been made”, “Yes, this is a real possibility”, or “No”.

Take note that we asked after “in-house reshoring” experiences or intentions thereto (see Gray et al. 2013 p. 28; Engström et al. 2018), and not after other forms of backshoring, like outsourced production to an overseas supplier that could be brought back to the home base via an internalization or insourcing action.

The base population for the survey consisted of some 2,000 firms that SPRI had recently interacted with due to expressions of interest or applications by these firms for innovation, R&D, and internationalization support programs offered by the Basque Government. As such, the base population may not be representative of the entire universe of firms in the Basque Country. However, since we are primarily interested in the adoption of Industry 4.0 technologies (related to innovation) and decisions in the realm of international business (related to backshoring), it can be argued that we targeted a relevant set of “likely instances” from which to source insights.

The survey was launched at the end of January 2019 and was closed in late February 2019, resulting in 475 valid answer sets. The answers from these 475 companies were screened to, firstly, retain the companies that engaged in production activities abroad through foreign manufacturing subsidiaries. A procedure that was also followed by Dachs et al. (2019)

Secondly, we considered only companies that operate in B2B markets and that are industrial in nature, being a:

- “manufacturer of finished products for other industrial firms,”
- “supplier of parts or components to other industrial users,”
- “system provider to other industrial users” or
- “provider of industrial services.”

Companies that are chiefly a distributor of goods or active in B2C markets were thus left out.

This led to a final sample of 63 companies able to make informed judgments on backshoring issues.^11^ In terms of number of employees, the sample has the following characteristics (see Table 3):

**Table 3:** Characterization of the sample in terms of number of employees

Number of employees	% of sample
Less than 10	0%
Between 10 and 49	13%
Between 50 and 249	46%
More than 250	41%

*N* = *63*

Similarly, Table 4 shows the main business activity of the sample members:

**Table 4:** Characterization of the sample in terms of business activity

Business activity	% of sample
Manufacturer of finished goods for industrial customers	54%
Supplier of parts or components for industrial customers	27%
Systems provider for industrial customers	10%
Provider of industrial services	10%

*N* = *63*

#### Case-based research activities

To attain research objective 3 (contextualizing the role of technology in backshoring decisions), we tracked companies that have already backshored manufacturing activities to analyze their experiences and motives. To identify such companies, we consulted the representatives of foreign offices that SPRI maintains across the world. This network of offices supports Basque businesses abroad, but also keeps track of Basque companies closing overseas factories. As such, it is a privileged observer of backshoring cases. Consultations with these representatives led to the identification of nine Basque companies with backshoring experiences.

In Table 5, essential features of these nine companies are presented.

**Table 5:** Basque companies with backshoring experience

Company	Approximate turnover in 2018 in million euros	Industry	Backshoring from/to
A	250	Electric power systems	Brazil to Spain
B	125	Bicycles	China to Spain (and Portugal)
C	200	Home appliances	Brazil to Spain
F	250	Kitchen equipment	China and Turkey to Spain
I	700	Vehicles	China and India to Spain
N	25	Metal mechanics	China to Spain
O	400	Electric power systems	Brazil and Turkey to Spain
S	100	Hand tools	Argentina and China to Spain
T	10	Casting technologies	China to Spain

*own elaboration*

Contact was established with the above companies and seven of them agreed to an on-site interview.^12^ The visits were held in late February 2019. In four cases, the interviewees were general managers, whereas in the other cases the contact person was the company’s international business director, a senior advisor to the company’s president and a chief technology officer, respectively. To prepare for the interviews, desk research activities were undertaken with regard to these seven companies, notably by going through company websites, corporate presentations, press articles and archives from SPRI.

On-site interviews were conducted in an inductive manner. We informed the interviewees of our interest in the role of Industry 4.0 technologies in relation to backshoring moves. However, we did not direct the conversations towards the role of technology in those processes. Instead, we followed an open approach, giving free rein to the speakers without limiting them through directed or closed questions. During these semi-structured interviews, which lasted between 60 and 90 min each, we “elicited” narratives from the company representatives. From a coding point-of-view, we first tried to comprehend what had led the companies to set up production activities in the foreign places from which they had meanwhile withdrawn. I.e., whether they pursued access to specific resources, whether they targeted cost-efficiency motives, whether they served market-seeking goals, and/or whether they went after strategic asset-seeking advantages (Dunning 1998). Next, we tried to find out why the company had backshored these production activities. For example, because it was not achieving the foreseen objectives, because the company had changed its (international business) priorities or strategy, because certain circumstances had changed and/or because the overseas branch plant had fulfilled its mission. To categorize the key factors behind the backshoring decisions, a taxonomy rooted in the OLI paradigm and Transaction Cost Economics was used (see Sect. 2.3). I.e., to digest our interviews we apply an “eclectic” framework made up of the three tiers from the OLI paradigm (Organization-specific, Location-specific and Internalization-specific advantages), and add the “behavioral uncertainty” and environmental uncertainty” components from TCE (Williamson 1985). We argue that this can help to provide relief to the reasons for backshoring compared to when these types of uncertainties are treated as (implicit) elements of internalization (dis)advantages or location-specific (dis)advantages.

As a follow-up to the interviews, a summary of the take-aways and our interpretation of the factors that played a role in the off- and backshoring decisions were presented to the respective companies to enhance a correct reflection of their experiences. This led some of the interviewees to propose modifications and additions, which helped to put each case into a more correct perspective (Creswell and Miller 2000).

## Findings

### Descriptive survey-based results

#### Adoption level of digital technologies

**Fig. 1:**
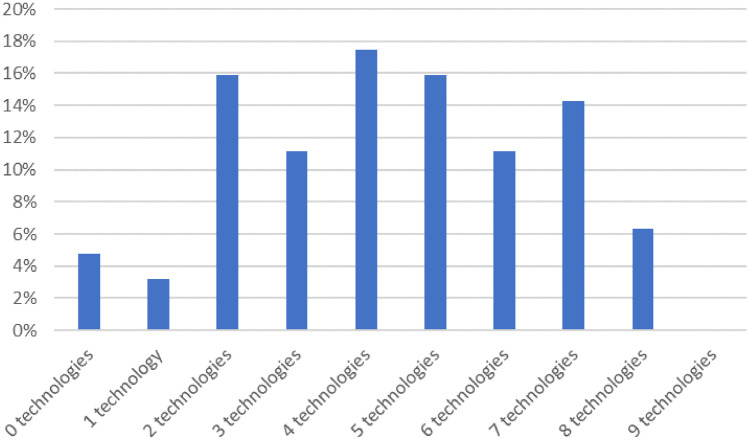
Adoption level of the nine technology pillars supporting Industry 4.0. *N* = 63

As regards the adoption of digital technologies on behalf of the 63 companies that make up our sample, we found the following:

With regard to the nine technologies that were considered, over 95% has adopted at least one of them and close to two-thirds of the companies had implemented four or more Industry 4.0 technologies (See Fig. 1).

Cybersecurity is the most widely implemented technology, with around 75% of the firms making use of it, followed by Internet of Things and Cloud computing. These two technologies have been implemented by close to 60% of the companies. The least implemented is augmented reality, with below 15% of the respondents making use of it. Robotics, big data analytics and 3D printing occupy middle positions, with some 45–50% of the respondents using these technologies.

In addition, if we look at the technology inventory that the DTM considers (see also Table 2),^13^ we conclude that approximately 98% has adopted at least two of them and close to 75% of the companies had implemented four or more technologies. Interestingly, the percentages are slightly higher than for the Industry 4.0 technology selection, which is arguably related to the fact that some technologies are more mature or lower entry (like automated machinery and social media). Indeed, automated machinery is implemented by 75% of the firms, whereas social media is adopted by more than 60% of the sample.

#### Interaction between adoption of digital technologies and backshoring decisions

Regarding the question whether adoption of the listed digital technologies may lead to backshoring maneuvers, 6% of the sub-sample agreed with this assertion and indicated that they had made concrete decisions to backshore production activities.^14^ Another 21% likewise agreed with this viewpoint, but from a forward-looking perspective, indicating that the adoption of Industry 4.0 technologies could make future backshoring decisions plausible. The rest of the respondents, 73%, opined that the adoption of digital technologies would not lead to backshoring decisions by their firm.

**Fig. 2:**
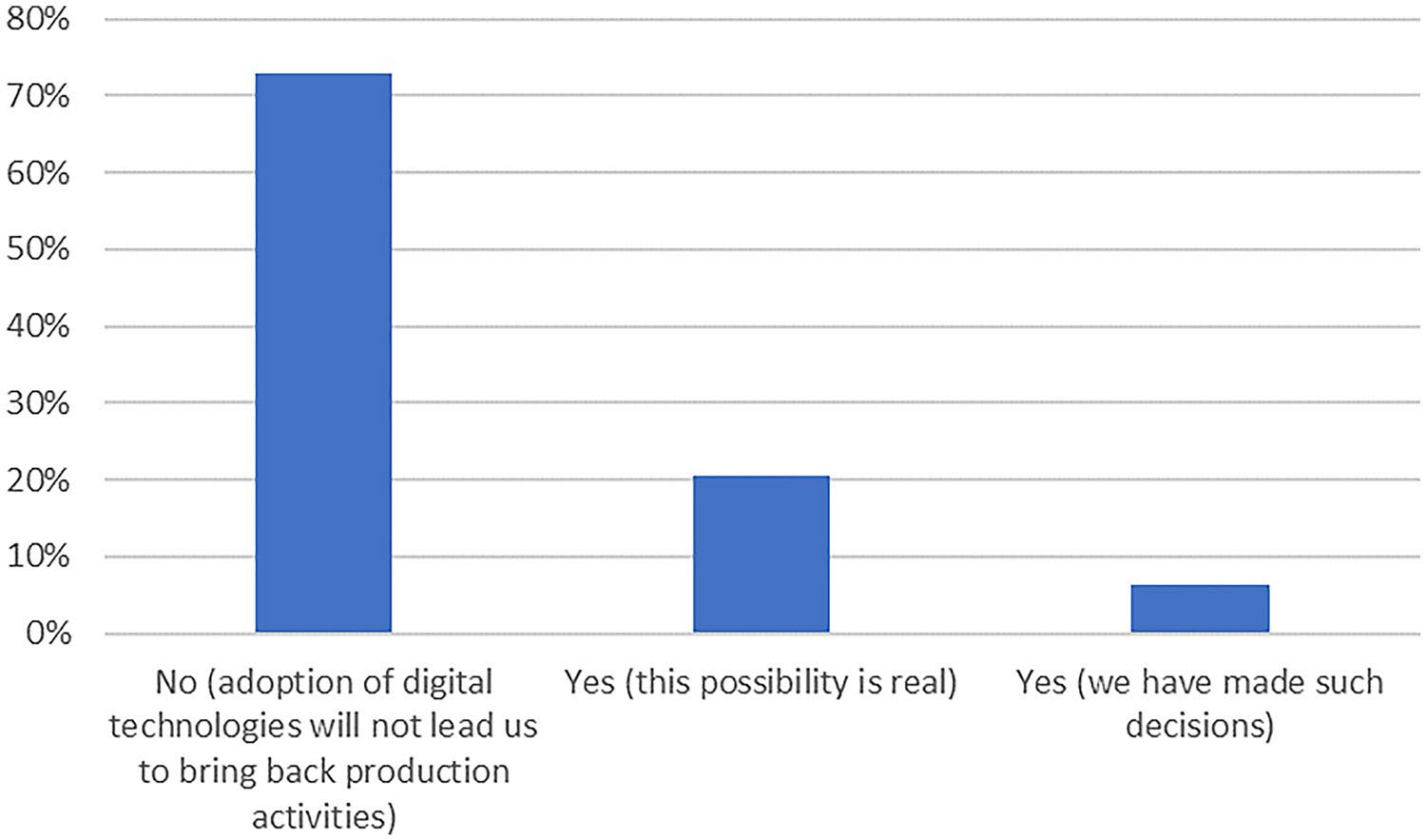
Expectations regarding the impact of adopting digital technologies on the backshoring of production activities. *N* = 63

**Fig. 3:**
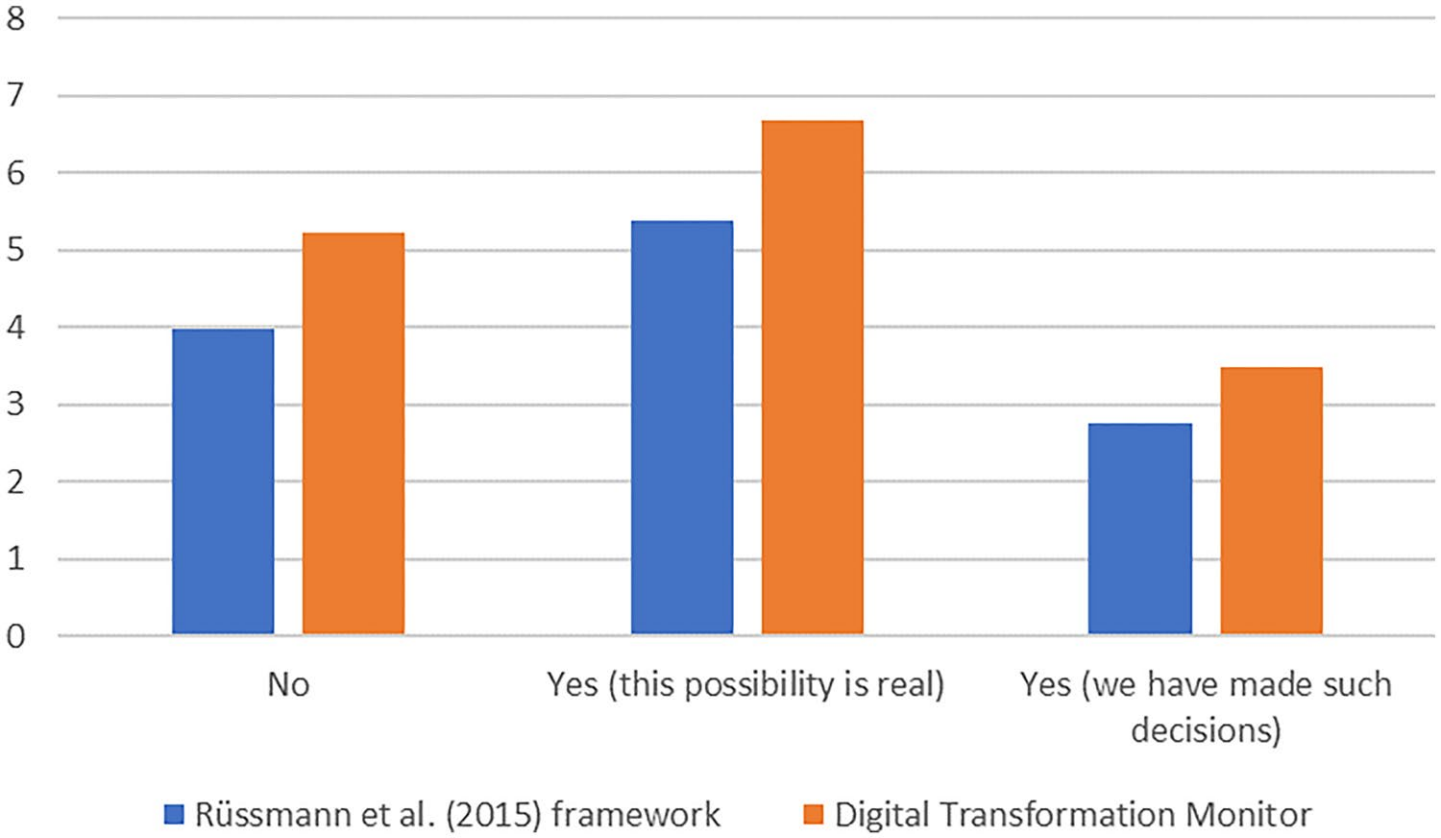
Number of digital technologies adopted in comparison to companies’ production backshoring postures. *N* = 63

In addition, based on the answers to the question of whether “the adoption of digital technologies may lead to backshoring maneuvers” (see Fig. 2), we looked at the average number of technologies from the Rüßmann et al. (2015) framework and the ones from the DTM inventory. This led to the following picture:

Figure 3 reveals that the firms that actually took the decision to backshore (answers on the right-hand side of the graph) are not the most prolific adopters of digital technologies. I.e., their adoption scores regarding the Industry 4.0 technologies according to Rüßmann et al. (2015) as well as from the DTM are lower than those for the group of firms that consider backshoring decisions to be a realistic prospect (answers in the center). Even more striking, their technology adoption scores are also outperformed by the group that does not expect to take production backshoring decisions (answers on the left-hand side of the graph).

Take note that while the technology adoption scores on the DTM inventory exceed those of the Industry 4.0 framework of Rüßmann et al. (2015), it is also true that the DTM considers 10 technologies, whereas Rüßmann et al. (2015) focus on 9.

### Regression results based on survey data

#### Testing at the aggregated level of the Industry 4.0 technology set

Using the respondents’ data on technology uptake and backshoring intentions / decisions for non-parametric testing led to the following results. Firstly, we looked separately at the data and scores for the three sub-groups (Yes, we have made backshoring decisions; Yes, this possibility is real; and No). Then, we carried out a One-Way ANOVA Kruskal–Wallis test. This revealed a significant relation (p = 0.036) between the adoption of Industry 4.0 technologies and backshoring (p-value threshold: below 0.05).

**Fig. 4:**
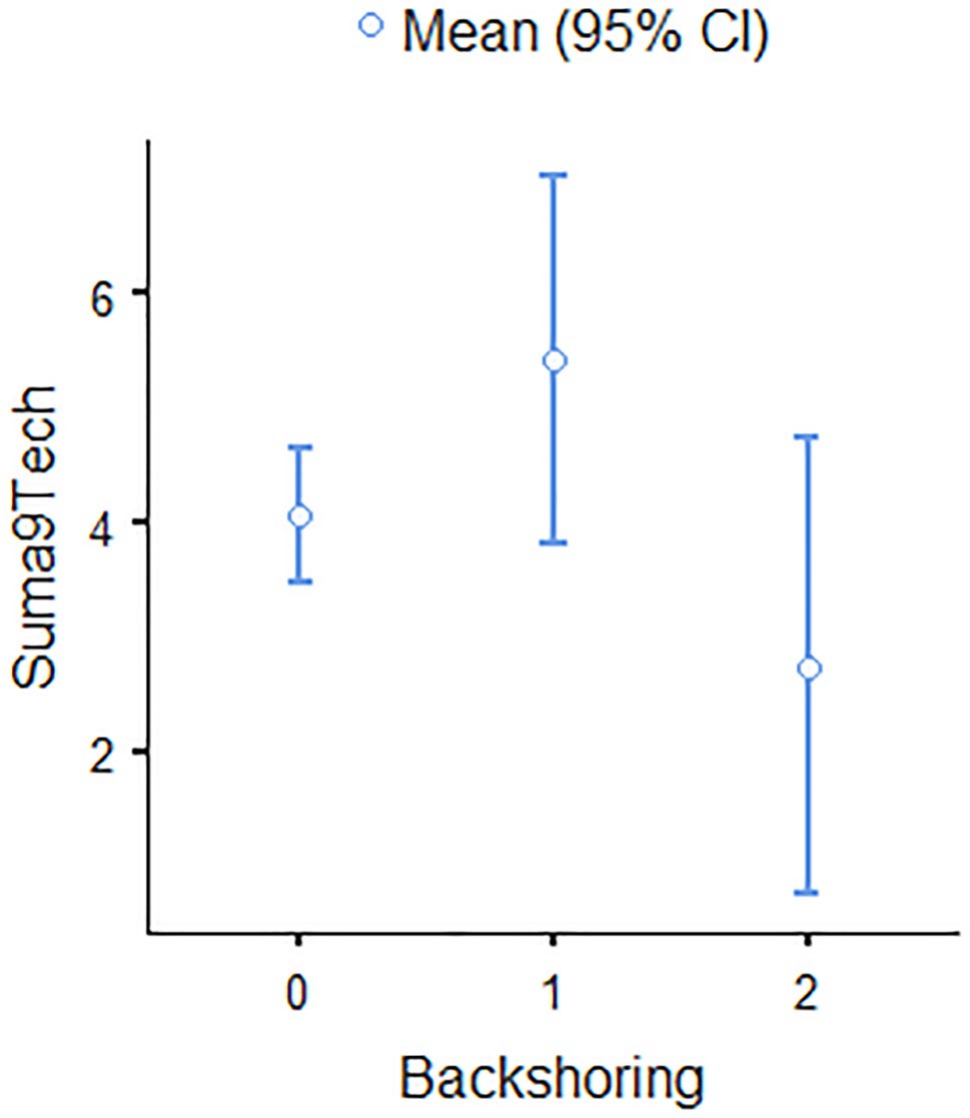
Plotting range of answers and average scores on Industry 4.0 technologies adoption versus backshoring postures based on Tukey post-hoc test. Source: own elaboration

A first check to see whether we actually were onto something, was to plot the data based on a Tukey post-hoc test (see Fig. 4). When doing this, it became obvious that there is a lot of overlap between the answer range of the firms without backshoring intentions (vertical bar 2) and the answer range of those with backshoring intentions (1) or who had taken decisions to repatriate activities in the (near) future (0). I.e., from a horizontal perspective, the vertical bars coincide partly. This basically implies that we can not affirm that the adoption of a larger amount of Industry 4.0 technologies changes’ companies posture towards backshoring and/or that it leads to a bigger chance on backshoring.

A further post-hoc test that we carried out was a -more granular and demanding- Dwass-Steel-Critchlow-Fligner pairwise comparison (p-value threshold: below 0.05). These comparisons neither backed up the previous Kruskal–Wallis tests. Rather, they weakened the signs of a significant correlation between the variables considered.

Dwass-Steel-Critchlow-Fligner pairwise comparison results.

Sub-group 0 versus sub-group 1 p = 0.091.

Sub-group 0 versus sub-group 2 p = 0.315.

Sub-group 1 versus sub-group 2 p = 0.157.

Afterwards, we regrouped the original three sub-groups into two. A first one containing the companies with backshoring intentions (Yes, this possibility is real) and effectively taken decisions towards the (near) future (Yes, we have made such decisions), and a second one made up by the companies that did not expect to backshore production activities (No). This regrouping was done to give some substance to the “Yes”-group, as with only 4 companies counting with firm backshoring decisions, comparisons on a trilateral basis would quickly become meaningless.^15^ The subsequent Welch One-Way ANOVA test also delivered a non-significant result (p = 0.372) regarding the relationship between the nine Industry 4.0 technologies and backshoring postures (p-value threshold: below 0.05).

Altogether then, the tests at an aggregated level for the Industry 4.0 technologies did not show a convincing relation with backshoring behavior of the sampled firms.

#### Testing the relevance of individual technologies for backshoring

Next, tests were conducted on the respective, individual, technologies that make up the Industry 4.0 technology set and whether the implementation of one or more of them is (significantly) more abundant among firms with backshoring intentions/decisions versus those without such intentions/decisions. This way, we avoid that a grouping of technologies blurs the sight on individual technologies that matter more than others for backshoring dynamics.

The individual tests revealed the following P values (threshold value: below 0.05) for the Industry 4.0 technologies:

1. Internet of Things: not significant (0.253)
2. Cloud Technologies: not significant (0.190)
3. Big Data Analytics: not significant (P = 0.800)
4. Virtual Simulation Systems: not significant (0.388)
5. Augmented Reality: not significant (0.136)
6. 3D Printing: not significant (0.836)
7. Horizontal and Vertical Systems Integration: almost significant (0.064)
8. Robotics: almost significant (0.071)
9. Cybersecurity: not significant (0.485)

In addition, we carried out the same tests for a series of additional technologies. This resulted in the following findings:

- Artificial Intelligence: not significant (0.815)
- Automated Machinery: not significant (0.707)
- Cyber-Physical Systems: not significant (0.202)
- Mobile Services: not significant (0.870)
- Social Media: not significant (0.148)

From the above results, it turns out that there are just two technologies that show signs of association with backshoring decisions: Horizontal and Vertical Systems Integration and Robotics. None of the others do.

#### Testing the relevance of individual technologies for specific backshoring rationales

Apart from asking the survey participants whether “the adoption of digital technologies may lead to in-house backshoring maneuvers”, we also asked the companies whether they thought that the use of the digital technologies they had implemented could lead to a reallocation of production activities via other ‘mechanisms’? I.e., by means of not/less offshoring of production, or by substituting foreign suppliers by national ones. The answers to this question serve as an indication of whether Industry 4.0 technologies can have indirect backshoring effects.

In response to this more broadly framed question, 49 companies indicated that they could foresee this kind of consequences from the adoption of Industry 4.0 technologies. The remaining 14 companies did not expect this to happen.

Against this backdrop, a follow-on question was posed to the sub-sample of 49 companies. They were asked in which way the adopted digital technologies would influence production location and supplier selection decisions?

As answer options they were presented a cost-oriented and a quality-oriented rationale. The first option referred to the ability of digital technologies to make production activities in the home base more cost-competitive compared to offshore locations. The second option referred to the ability of digital technologies to improve the quality of production processes and of tying production steps together, diminishing the interest in delocalizing or fragmenting production steps to/across foreign places.

Take note that companies were able to tick multiple options. That is: they could argue in favour of one of the rationales presented to them or to both. As regards the respective rationales, the firms were asked with which extent these applied according to them (1: very strongly, 2: strongly, 3: moderately, 4: in a limited manner or not at all).

Next, we focused on the companies that attributed a 1 or 2 to either one of the rationales, and reviewed the respective technologies that they had adopted. As such, we check whether specific technologies stand out for specific production strategy rationales. I.e., we group the answers according to a cost-oriented and a quality-oriented rationale or production strategy, and then look at the technologies adopted by the respondents from the respective groups.

Consequently, the following P values were obtained (threshold value: below 0.05).

Firstly, we present the results on the cost-oriented rationale for the nine Industry 4.0 technologies:

1. Internet of Things: not significant (0.451)
2. Cloud Technologies: not significant (0.647)
3. Big Data Analytics: significant (0.047)
4. Virtual Simulation Systems: not significant (0.938)
5. Augmented Reality: not significant (0.644)
6. 3D Printing: not significant (0.938)
7. Horizontal and Vertical Systems Integration: 0.831
8. Robotics: not significant (0.830)
9. Cybersecurity: not significant (0.814)

In addition, the same assessment was made for the supplementary technologies

- Artificial Intelligence: not significant (0.414)
- Automated Machinery: not significant (0.339)
- Cyber-Physical Systems: not significant (0.707)
- Mobile Services: not significant (0.938)
- Social Media: not significant (0.181)

From the above it follows that the only technological pillar that companies attribute an important role to for improving the cost-competitiveness of home-based production operations (and influencing production retention decisions and/or local supplier selection positively) is big data analytics.

Secondly, when examining the quality-oriented rationale we get the following results for the nine Industry 4.0 technologies:

1. Internet of Things: not significant (0.165)
2. Cloud Technologies: not significant (0.647)
3. Big Data Analytics: not significant (0.459)
4. Virtual Simulation Systems: not significant (0.938)
5. Augmented Reality: not significant (0.193)
6. 3D Printing: not significant (0.938)
7. Horizontal and Vertical Systems Integration: not significant (0.718)
8. Robotics: not significant (0.830)
9. Cybersecurity: not significant (0.814)

The same assessment for the supplementary technologies from Table 2 yields the following results:

- Artificial Intelligence: not significant (0.962)
- Automated Machinery: not significant (0.339)
- Cyber-Physical Systems: not significant (0.707)
- Mobile Services: not significant (0.580)
- Social Media: not significant (0.938)

Consequently, this test does not reveal any technology that companies who are driven by quality-oriented motives for their location decisions adopt more systematically than those with an opposite posture.

### Case-based research results

#### The role of technology in backshoring decisions

As can be seen in Table 6, the companies with previous backshoring experiences reveal nowadays a rather pronounced technology adoption profile. The average number of technologies adopted from the Industry 4.0 framework is 4.57 (higher than the average for effective backshoring firms shown in Fig. 3) and 2.43 for the additional technologies from Table 2).

**Table 6:** Adoption of digital technologies among case companies with previous backshoring experience

Industry 4.0 technologies	Company A	Company B	Company F	Company I	Company N	Company O	Company S
Internet of Things	X		X	X		X	X
Cloud Technologies	X			X		X	
Big Data Analytics	X			X		X	
Virtual Simulation Systems				X			
Augmented Reality							X
Additive manufacturing / 3D Printing	X	X				X	X
Horizontal and Vertical Systems Integration	X		X			X	X
Robotics	X	X	X	X		X	X
Cybersecurity	X	X	X			X	X
**Additional technologies**							
Artificial Intelligence				X			X
Automated Machinery	X	X	X	X	X	X	X
Cyber-Physical Systems							
Mobile Services	X	X	X		X		
Social Media	X	X	X	X		X	X

Own elaboration based on inputs from companies

As such, this might be somewhat at odds with the results from Sect. 4.2. However, practically all of the backshoring cases we review took place before the adoption of Industry 4.0 technologies came on steam, so the role of these specific technologies certainly has to be relativized. Moreover, past backshoring behavior on behalf of specific firms does not imply that these same companies will repeat such decisions, even if they maintain (several) production plants overseas (which is the case for all case companies except for Company N). In fact, five of the companies with previous backshoring experience participated in our survey and all of them indicated that they did not expect to undertake further backshoring actions. Hence, their answers form part of the “no firms” in Figs. 2, 3 as well as the subsequent regression analyses.

The seven firms that were interviewed provided insights into 11 backshoring cases where overseas plants of Basque companies were closed or saw their production activities drastically reduced through a transfer of manufacturing activities to the home base.^16^ Five of the firms had undertaken a single backshoring operation, whereas two others had backshored twice. In Table 7, we set out the details of the respective experiences.

**Table 7:** Specifics on backshoring cases

Company and country where it set up production	Year of opening / acquisition of plant and year of closure / sell-off / reduction of activity	Entry mode	Why offshore?	Why backshore?
**Company B-China** **Company F-China** **Company I-China**	2007–2015 2007–2015 1995–2012	Greenfield FDI Greenfield FDI Greenfield FDI	Expectation that the Asian market for premium bicycles, robust kitchen equipment and high-end buses would grow “Market-seeking” Company B and F form part of an industrial cooperative and together with several fellow cooperative firms these two companies decided to set up shop in an industrial park that would only shelter affiliates of this Basque cooperative “Bandwagon effect”	Companies B and I targeted premium market segments whose sales results in the Chinese and adjacent markets remained below expectations Company F’s products did match local pockets of demand, but the company found it hard to stand out among local producers of similar products, so the lack of competitive advantage or the lack of appreciation for its superior value caused it to lose ground in the market Consequently, the plants of companies B and F ended up producing for export markets, although they were intended to produce for local markets
**Company N- China**	2004–2015	Greenfield FDI	Chop up the value chain by locating design activities in Europe and production activities in China. Seize cost advantages through offshoring manufacturing “Efficiency-seeking”	The Chinese plant of Company N produced a lot for the European and the North American market Over time, manufacturing in the Eurozone became more attractive, as producing in China started to get more expensive. Also, from a trade perspective, it became of increasingly greater interest to produce within the Eurozone, as the United States taxes imported bearings from China more heavily than when they are produced in Europe Company N also pointed out internal financial and investment issues: due to a lack of resources to keep the multinational structure that the firm had created intact, it was forced to reconsider its international strategy and overseas production activities Similarly, as the company opted for more R&D-intensive and high-end products, it decided to pull back into its home base and concentrate its activities in the Basque Country
**Company S-China**	2008 to present (although production has been transferred to Europe)	Greenfield FDI supplemented by takeover of local player with multiple plants	Company S merged with peers from Northern Europe and the United States. The joint holding counted with an own Chinese factory and went on a take-over spree in China. The outcome was an ensemble of plants in China and a sub-optimal distribution of “which plant produces what for which markets” “Strategic asset-seeking”	In this case, restructuring and rationalization of the global production apparatus and the respective “origin” (where they produced)– “destination” (which markets they supplied) relations constituted the trigger for backshoring operations This meant that Company S decided that part of the Chinese production for the Northern European market would be manufactured within Europe This was done for reasons of both logistics and quality, but also since the advanced adoption of new production technologies at European production sites made them more cost competitive than before
**Company I-India**	2001–2015	Joint venture with two local partners, making use of existing production facilities at first and adding a greenfield plant in 2010	Expectation that the Asian market for high-end buses would grow “Market-seeking”	Company I suffered from a lack of appreciation of its premium products and high-safety buses, as well as disappointing forecasts for the high-end coachwork segment in the Indian market Admittedly, the firm may not have planned the market entry very well, in the belief that having a production presence in the (BR)IC countries was a must and being advised by third parties that the growth possibilities for its product line in the Indian market would be substantial
**Company F-Turkey Company O-Turkey**	2006–2016 1999–2006	Greenfield FDI Greenfield FDI	Sales potential on the domestic (hotel) market “Market-seeking” Relatively well- skilled human resources and supply chain “Resource-seeking”	Company F and Company O blamed turmoil in the domestic (Turkish) market—both from an institutional point of view and as regards sales forecasts—for their decisions to withdraw Both also suffered from a lack of appreciation for their products by local customers, who generally showed a low willingness-to-pay and a tendency to settle for copycat products On top of this, both Company F and Company O indicated that their products manufactured in Turkey were not suitable for other markets, reducing the possibility of fitting the Turkish plant into their overall international business strategy Over time, declining sales meant that maintaining a dedicated plant for the Turkish market became unsustainable for both companies
**Company A-Brazil** **Company O-Brazil**	2003–2017 (sell-off to a competitor) 2005–severe reduction in activities since 2016	Take-over of existing local company Greenfield FDI	Take-over of diverse kernels of knowledge and technology bases for subsequent transfer and roll-out across the globe “Strategic asset-seeking” (Company A only) Entry to seize sales potential on domestic market “Market-seeking” (both companies)	Both cases suffered from a worsening of domestic market conditions and sales outlooks At the same time, the Brazilian plants were faced with growing environmental uncertainties, including: union pressure, tariffs on parts imports, ineffective functioning of local (segments of the) supply chain The two companies characterized Brazil as a challenging country, administratively speaking, with a lot of red tape and a highly politicized business life The subsequent institutional uncertainties made it unattractive to continue producing in Brazil Furthermore, Company A and Company O pointed to a lack of strategic fit between their Brazilian products/manufacturing activities and their evolving core business and international business activity. In the end, the Brazilian activities became increasingly disconnected from the companies’ product and technology portfolio, leading to a phasing out of Brazilian production operations
**Company S-Argentina**	1963 to present (although production has been transferred to Europe)	Greenfield FDI	Company S merged with peers from Northern Europe and the United States. While Company S already had a branch plant in Argentina since decades, the joint holding also took over an additional factory in Argentina. The outcome was a redundancy in production capacity and a sub-optimal distribution of “which plant produces what for which markets” “Strategic asset-seeking”	Similar to the experience of Company S in China, the Argentinian backshoring case is a consequence of restructuring and rationalization of the company’s global production apparatus and its respective “origin”– “destination” relations Within this context, it was decided that the Argentinian production for the US market would be manufactured in Europe The decision to transfer US-bound production out of Argentina was based on logistics, quality and cost arguments. As far as cost reductions were to be gained from this transfer, they were fueled by the superior level of automation and technological progress at the European plants compared to the one in Argentina

Own elaboration based on interviews and desk research

In five cases the country from which production activities were backshored was China, whereas there were two backshoring experiences from Brazil and also two from Turkey. In addition, there was one backshoring case from India and one from Argentina.

On average, the time that the overseas plants were operational until backshoring decisions were taken was 15 years, although this number lowers to 11 years if we do not consider the Argentinian plant of Company S. This plant forms an outlier as it was created in 1963 and had been producing for over 50 years before it devolved production activities to the Basque Country via backshoring decisions taken by its owner.

Building on the previous table, it turns out that disappointing sales in the markets that offshore factories were intended to supply was typically the motivation for backshoring (Company A, B, F and O). In the case of Company B and F backshoring was also due to erroneous forecasts of the offshore market sales potential. Company I also suffered from this. In the case of Company S, a difference in technological sophistication of manufacturing in Spain compared to the technology level of Company S’ plants in Argentina and China did function as a catalyzer for backshoring decisions. Particularly as this translated into a cost disadvantage for the Argentinian and Chinese plants versus the Basque plant. Hence, through intra-firm competition for production orders, the Basque factory was assigned manufacturing activities that used to be carried out in either Argentina or China. Company N also testified that as they were facing (labour) cost increases at their Chinese plant, this formed a trigger for backshoring. Problems with product quality was mentioned by Company N and S. Too long delivery times and logistics complexity to serve the end markets also resounded in the case of Companies N and S, as their offshore plants produced for clients on other continents. Eventually, this also played a role for Companies B and F as their offshore plants shifted their focus from selling on-site to producing for Europe. In a similar vein, companies A, B, F, I and O suffered from an overengineered or too high-end product offer for the markets they were supposed to serve. Hence, their plants gradually lost their reason for being in the countries they were located. Lack of ability to manage and integrate the overseas activities properly with the rest of the company’s business, was mentioned by Company N. Additionally, Company N suffered from financial constraints. Finally, contextual factors like trade barriers, taxes on products manufactured in China to be shipped to other continents, tense industrial relations and political instability played a role in the backshoring decisions of Companies A, F, N and O.

## Discussion

### Level of digital technology adoption

Overall, the 63 companies from the survey sample reveal a considerable level of digital technology adoption. If we compare our findings with those of BCG (2016), it seems fair to state that our sample is reporting a considerable adoption rate for Industry 4.0 technologies. That is, BCG (2016) established that 19% of some 300 firms from Germany and 16% of around 300 US firms had either implemented a full Industry 4.0 concept (such as running a smart factory) or initial measures toward this concept (such as the introduction of autonomous robots). Similarly, Ancarani and Di Mauro (2018, pp. 5–6) and Ancarani et al. (2019, p. 367) come to an adoption percentage that is in line with the results from BCG (2016). Against this backdrop, the adoption of robotics, automated machinery or horizontal and vertical system integration on behalf of our sample easily doubles those percentages.

### Frequency of backshoring

Our research revealed 13 backshoring experiences on behalf of 9 firms. On a total of 666 foreign plant locations held by Basque firms overseas (SPRI data from 2019), these 13 backshoring cases represent 2.0%. Similarly, if we compare the fact that there are an estimated 296 Basque firms (data from SPRI) that have one or more overseas production plants and only 9 of them have undertaken backshoring, this represents a maximum of around 3.0% of the total.

This range of backshoring experiences is quite in line with the 4.3% that Dachs et al. (2019, p. 4) establish based on data from the European Manufacturing Survey. It is also in line with the percentage that can be reconstructed from Johansson et al. (2019). They identify 160 backshoring projects out of 4601 plant locations, representing 3.5%.

### Interdependences between digital technology adoption and backshoring decisions

When comparing the survey results on digital technology adoption with findings from other sources (see Sect. 5.1), it seems fair to state that our sample is characterized by a pronounced uptake of digital technologies. However, the survey respondents provide mixed results when it comes to backshoring decisions and intentions towards the (near) future.

Whereas a small percentage (± 6%) of the participants declared to have taken decisions with a view to repatriate activities, there was a larger group (± 20%) who indicated that they may take such decisions. Still, the lion share of companies asserted that they would not take backshoring actions.

While the Dwass-Steel-Critchlow-Fligner pairwise comparisons revealed no significant correlation between backshoring and Industry 4.0 technologies adoption, it is interesting to note that the ones with future intentions to backshore display the highest digital technology adoption rate (see Fig. 3). Therefore, if we relate our results to the following statement of Ancarani et al. (2019, p. 367): “The data collected suggest that the diffusion of Industry 4.0 among companies backshoring to Europe is not widespread (14%)”, they provide rather ambiguous support. On the one hand, the participants to our survey who have decided to backshore in the (near) future score lower on digital technology uptake than those who consider taking backshoring decisions and those who do not plan any backshoring operation. As such, this appears to be in line with the above statement. On the other hand, precisely as our survey data indicate that the firms who consider backshoring as a future option are the ones with the highest adoption rate of Industry 4.0 technologies implies that future cases of backshoring could be carried out by firms with a pronounced Industry 4.0 profile.

Moreover, even if the implementation of Industry 4.0 technologies does not seem to be the root cause for backshoring dynamics, it can strengthen them. The fact that almost 20% of the surveyed companies indicated that the use of digital technologies turns ‘taking backshoring decisions’ into a real possibility (versus only 6% of the survey participants with firm backshoring decisions), could point in this direction. It would also hint at a trend rupture (in line with Dachs et al. 2019) between past and prospective backshoring. In support of this trend rupture idea one can argue that the adoption of digital technologies is a recent phenomenon and its effects on production and plant location decisions can only become visible with time.

### The role of individual digital technologies for backshoring

When assessing whether specific technologies hold a relationship with backshoring attitudes, we find that robotics and horizontal and vertical systems integration are the only ones that reveal a nearly significant correlation with backshoring. The finding regarding robotics is in line with postulations of Ancarani and Di Mauro (2018) and Fratocchi and Di Stefano (2019). Studies that have focused on the individual role of horizontal and vertical systems integration vis-à-vis are not available, but our finding makes sense from an embeddedness perspective. That is: the fact that this technology -via its horizontal dimension- has an inter-firm character that implies coordination with surrounding companies, could lead to backshoring activities to places where the focal firm interacts closely with suppliers and (lead) users (Forsgren et al. 2005).

When checking whether robotics and automated machinery, on the one hand, and 3D printing and Cyber-Physical Systems, on the other, are more pronouncedly adopted by firms that adhere respectively to a cost or quality production strategy, our data do not provide back-up to the postulations that Ancarani and Di Mauro (2018) and Fratocchi and Di Stefano (2019) issue. While this does not exclude that certain technologies can be useful from a cost reduction or quality improvement rationale (or a pursuit of raising flexibility, delivery or customization levels, for that matter), one should also realize that their usefulness will depend on the type of production activities to be backshored. I.e., if large scale production activities are brought back to the home base, the chance that 3D printing will play a crucial role is not so big. In fact, even when looking at firms that manufacture high-end or premium quality products (like Company I and O from our case studies), its relevance may be reduced and it may be largely limited to (rapid) prototyping or the production of accessories and optional features.

### Contextualizing the role of technology in backshoring decisions

When looking at the findings from the historic backshoring cases, technology does not appear to have acted as a meaningful driver for the repatriation decisions taken. Take note that this goes for technology in general and not for specific Industry 4.0 technologies in particular, as most of the backshoring decisions by our case companies were taken at a time that Industry 4.0 technologies were not around. Only in the case of Company S did the difference in technological sophistication of manufacturing in Spain compared to the technology level of Company S’ plants in Argentina and China contribute to backshoring decisions. Beyond the experiences of Company S, it seems that other factors played a more determining role when the reviewed companies took their backshoring decisions (see Table 8).

**Table 8:** Classifying backshoring motives according to OLI-TCE scheme

	Organization-specific disadvantages	Location-specific disadvantages of offshore production sites						Behavioral uncertainties		Environmental uncertainties	Internalization disadvantages	
Company	Product–market mismatch	Eroding sales in markets that the offshore site is intended to serve	Lagging production technology level compared to plants elsewhere (notably in the home base)	Rising cost level	Quality issues	Distance to (unforeseen) end markets	Distance to corporate R&D or design assets	Erroneous forecasting	Predictability of local staff behavior and performance	Institutional uncertainty (political instability, turbulent industrial relations, trade barriers)	Rationalizing product(ion) portfolio across manufacturing sites	Resource shortages (finance, staff) to coordinate foreign operations
A	X	X						(X)		X	X	
B	X	X				(X)		X				
F	X	X				(X)		X		X		
I	X							X				
N				X		X				X		X
O	X	X						(X)		X	X	
S			X	X	X	X					X	

*Own elaboration*

To start with, Companies A, B, F, I and O seemed unable to exploit their “organization-specific advantages” in the sense that they discovered ± to be offering the right product to the wrong market. That is, they ended up offering over-engineered products and/or were targeting marginal market segments in the countries they had set their sights on. This type of product-market mismatch as an ingredient for backshoring has also been referred to by Di Mauro et al. (2018).

When considering “location-specific (dis)advantages”, beyond the mentioned technology differential that Company S referred to, the following factors came to the surface. Rising cost levels as a sign of fleeting advantages of offshore locations (Bals et al. 2016) were reported by Company S, allowing its Basque plant to take over production assignments from its counterparts in Argentina and China. Company N also reported this, which -together with financial shortages that it was suffering from after the credit crunch- prompted it to divest of its Chinese plant and backshore its production activities. Quality issues were only declared by Company S as a reason for backshoring (Stentoft et al. 2016a, b). The distance and logistics to end markets became an issue for Companies B and F as a lack of success on their host markets led them to manufacture products for export to Europe. As this was an unforeseen downside, it influenced their backshoring decisions. As such, the argument that backshoring can serve to bring production closer to the end market (Johansson and Olhager 2018) applies to Companies B and F.

In terms of “behavioral uncertainties” we see that Companies B, F and I were forced to correct or revoke their prior internationalization decisions” (Kinkel and Maloca 2009; Dachs and Kinkel 2013) due to erroneous or overoptimistic forecasting. Also Companies A and O were confronted with deteriorating or disappointing sales in the markets that their offshore factories were intended to supply, and this motivated their backshoring decisions. Ultimately, these decisions can be related to the fact that market-seeking motives formed the primary motive to go abroad for the companies considered. Behavioral uncertainties in terms of deficient or opportunistic behavior of staff or suppliers was not explicitly reported.

“Environmental uncertainty” (e.g. in the form of institutional instability, political turmoil, tense labour relations) played a role in the backshoring decisions of Company A and Company O out of Brazil, and Company F and O out of Turkey. By extension, Company O argued that plant closures tend to be linked to moving out of unstable (commercial, political) markets rather than to production (technology)-related motives. Company N, for its part, referred to rising trade barriers hampering it to ship products from China to other continents in a competitive manner. This type of changing exogenous circumstances as a reason for exiting foreign investments align with findings from Anderson and Gatignon (1986), as well as Gylling et al. (2015), Mugurusi and De Boer (2014) and Barbieri et al. (2018).

As for “internalization disadvantages”, Company N reported a lack of coordination and oversight capacities as a catalyzer for backshoring, which resonates with findings from Brouthers and Nakos (2004), Müller et al. (2017) or Dachs et al. (2019). Rationalizing product(ion) portfolios across internal manufacturing sites and optimizing was an issue for Company A, O and S. As such, for them backshoring was also the outcome of a deliberate strategic intent. In the case of Company A, their production was increasingly spread out across the world (particularly in South America) to comply with local content rules in different markets. Recently, they decided to divest several of their earlier acquisitions as the products that these plants manufactured were insufficiently marketable across the globe, making them less interesting from a strategic business perspective. Ultimately, this led to the sell-off of Company A’s Brazilian subsidiary. This aligns with the observation of Di Mauro et al. (2018) that if products manufactured in offshore locations end up being poorly adjusted to the owner’s portfolio, this can give rise to a firm’s withdrawal from such production places. In the case of Company S and its plants in China and Argentina, the transfer of production to Europe clearly reflects the desire to streamline the product–market combinations the respective plants serve. Similarly, Company O argued that they scrutinize their global production apparatus regularly and try to move to places of strategic importance and where there is a stable and sizeable market available. This is a kind of “strategic asset retention” behavior, which certainly in the case of Company S and A aligns with their strategic asset-seeking motives to create or acquire foreign production capacity in the first place. As such, this is in line with assertions by Grandinetti and Tabacco (2015) or Robinson and Hsieh (2016) that global reorganization rounds can unlock backshoring movements.

Altogether then, technology seems to form part of the backshoring equation, but it certainly did not appear to constitute a preponderant variable. Accordingly, during the company interviews, it became clear that if backshoring takes place, it is often due to multiple factors. And when backshoring is being considered, (digital) technologies can act as a catalyst or accelerator for a backshoring decision that made sense all along. Hence, in the cases under review seemed to have been a catalyzer, rather than an instigator, trigger or driver.

## Implications

### Conceptual implications

It seems fair to state that while a growing body of backshoring research is taking shape, including research that looks at it from an Industry 4.0 perspective, the range of technologies considered in such studies tends to be small. In furtherance to Stentoft et al. (2020), the present study underlines that reviewing companies’ adoption of digital technologies can and should be expanded to understand the dynamics between Industry 4.0 and backshoring. One, because there is more to Industry 4.0 than just two or three of the most adopted digital technologies among manufacturing firms. Two, to paraphrase Gray et al. (2013), because it is important to define what Industry 4.0 is, which digital technologies go underneath this umbrella term, and how their uptake might evolve. These issues matter to avoid that Industry 4.0 is considered as a black box or that (backshoring) analysis in relation to Industry 4.0 focus on mere “digital technology mixes”. Consequently, breaking Industry 4.0 down into concrete technologies and assessing the joint and individual relation of the respective technologies with backshoring offers analytical surplus value.

In addition, the present paper’s consideration of behavioral and environmental uncertainties (from the TCE toolbox to predict governance arrangements) as separate categories next to the classic trichotomy of OLI (dis)advantages to foreign market entry/exit decisions (instead of treating them as parts of location-specific or internalization advantages) served to break further conceptual ground. I.e., it helped to segment the motives behind backshoring decisions in a more granular manner and expand the variety of factors or drivers, as well as bundles of them, affecting backshoring operations (Dachs et al. 2019; Johansson et al. 2019).

### Practical implications

The impact of Industry 4.0 technologies on backshoring operations is not clear-cut. While we did not find significant evidence that the adoption of digital technologies has stimulated backshoring so far, in the future this may happen. At least, the stated evidence we obtained on possible future backshoring in association to Industry 4.0 clearly supersedes the revealed evidence on past and present backshoring in relation to the adoption of digital technologies. Furthermore, happenings like the outbreak of COVID19 could foster additional backshoring activity (Barbieri et al. 2020; Strange 2020; UNCTAD 2020). Conversely, it is also possible that making supply chains more robust could be their preferred response to continue reaping the advantages of international trade and global value chains (Miroudot 2020). In fact, precisely Industry 4.0 technologies like Big Data Analytics can be leveraged to deal better with supply chain risks (Birkel and Hartmann 2020). Moreover, the idea that nearby sourcing will bring more stability and certainty is not so evident. In this regard, a recent OECD (2020) study highlights that in addition to higher costs, recurring to local sourcing can lead to higher volatility in securing supplies, as there are fewer channels for adjusting input provision. Such insights would then neither make it likely that we will witness some kind of de-globalization process nor that Industry 4.0 technologies would drive such a process forward.

In a similar vein, we obtained indications from our survey that the implementation of digital technologies may have a stronger dissuasive effect on possible offshoring decisions than on the inclination of firms to backshore production activities. This aligns with results from Müller et al. (2017) and Stentoft et al. (2020). Consequently, policy makers may have more interest in pursuing production retention initiatives than in creating backshoring agencies. The variety of factors in play for backshoring, as revealed by our case analyses, also hints at the importance of broader framework conditions compared to the adoption of digital technologies itself. This implies that place-based cluster and value chain development as part of industrial policies may also be important tools to foster backshoring (Nujen and Halse, 2017). Take note that the relevance of ecosystem quality in the home base is also echoed by Tate (2014), Baraldi et al. (2018) and Di Mauro et al. (2018). Consequently, backshoring firms should seek out places where an adequate supply chain and technological milieu is available for the production activities to be repatriated.

## Limitations and suggestions for future research

Evidently, the present paper does not come without limitations. First, our research has been based on a relatively small number of company observations from a restricted geographical area. As such, the possibility for generalizing our results is reduced.

Second, the fact that we worked with a reduced sample also generated methodological constraints. I.e., given that our survey only counted with four companies with firm backshoring decisions led us to combine this sub-sample with the one for companies with future backshoring intentions. While this was done with the purpose to enable the statistical analyses that we presented in Sect. 4.2, it reduces the purity of our comparisons.

Third, our case studies on companies with backshoring experience to assess the role of technology behind such decisions has limitations for appraising the relevance of Industry 4.0 technologies amidst those decisions. That is, the reviewed backshoring experiences took place during a period (2006–2017) in which Industry 4.0 and the uptake of corresponding digital technologies was largely absent. This implies that they only serve to assess the role of technology at large, but not of specific Industry 4.0 technologies, for backshoring. Additionally, it can be argued that instead of our mixed method of combining a questionnaire-based survey with case studies, full-fledged case studying could reveal more insights into this question.

Fourth, for a couple of the independent variables, we recurred to proxies that can certainly deviate from the original concepts in the Industry 4.0 framework we adhered to. Although this is common and also applies to other publications, it shows that it is important to set common definitions for concepts like Industry 4.0 and to operationalize its underlying technological building blocks in order to come to research results that are comparable and interoperable with other analyses. At present, such lack of commonality hampers the comparability of our findings with other studies.

The former are lessons to take into account for future research. In addition, we deem that further testing of which Industry 4.0 technologies matter for respective types of backshoring rationales or production strategies (as per Ancarani and Di Mauro 2018, and Fratocchi and Di Stefano 2019) is indicated.

Furthermore, looking at the adoption of Industry 4.0 technologies amidst other factors that play a role in decision-making around backshoring deserves following. Either by looking at practice-based bundles of relocation drivers (Johansson et al. 2019) or through a conceptual lens to such processes, like TCE and the OLI paradigm (Dachs et al. 2019; Johansson et al. 2019).

Finally, similar to how we treated behavioral and environmental uncertainties next to the classical OLI pillars, other central components from TCE like asset specificity of foreign investments or frequency of interaction between an offshore site and corporate Headquarters (closely related to coordination costs of foreign operations) may receive separate attention in backshoring studies.

## Data Availability

(data transparency): yes.

## Footnotes

1 It considers the following nine technologies as “endogenous” to Industry 4.0: Internet of Things, Cloud technologies, Big data analytics, Virtual simulation systems, Augmented reality, Additive manufacturing / 3D Printing, Horizontal and Vertical systems integration, Autonomous robots and Cybersecurity.

2 Gray et al. (2013) recognize 4 reshoring options in function of whether the original off-shored activity was performed in-house or outsourced and whether the back-shored activity will be performed in-house or by third parties. In the present paper, we focus on what they call “in-house reshoring”.

3 These are: Additive manufacturing, and Digital exchange of data with suppliers / customers in combination with Near real-time production control systems as a proxy for Horizontal and Vertical Systems Integration.

4 I.e., Product-Lifecycle-Management Systems, Systems for automation of internal logistics, Mobile/wireless devices to provide services, Technologies for human–machine interaction, and Digital visualization.

5 Robotics and 3D printing adhere to the Rüßmann et al. (2015) framework, whereas automated machinery falls aside.

6 That is: 3D printing and Robotics, whereas Automated machinery and Cyber-Physical Systems fall aside.

7 Take note that Stentoft et al. (2020) do not look at backshoring per sé, but at relocating production activities abroad.

8 See Table 2.

9 OLI stands for: Organization-specific, Location-specific and Internalization advantages.

10 Due to the COVID-19 pandemic, attention has grown for the disadvantages and risks that disruptions in (long haul) supply chains may suffer (OECD, 2020).

11 These 63 companies represent around 20% of the totality of Basque companies with overseas production plants (see Sect. 5.1).

12 Companies A, B, F, I, N, O and S were visited.

13 Big Data Analytics, Cloud Computing, Cybersecurity, Internet of Things, Automated Machinery, Robotics, 3D Printing, Artificial Intelligence, Mobile Services and Social Media.

14 Take note that the companies which declared that they have taken backshoring decisions referred to decisions in the here and now. So, recently taken decisions to come into effect in the (near) future. That is: around the time of the survey they had taken such decisions, but the repatriation of activities was to take place in future tense.

15 Off course, the decision to combine “Yes, indeed” and “Yes, maybe” also has its drawbacks, and this is something that we acknowledge.

16 In one case, the backshoring operation had a dual character, bringing one part of the production activities back to the Basque Country and another part to Portugal (nearshoring).

## Code availability

(software application or custom code): no.
